# How does pollen production of allergenic species differ between urban and rural environments?

**DOI:** 10.1007/s00484-023-02545-w

**Published:** 2023-09-02

**Authors:** Johanna Jetschni, Markus Fritsch, Susanne Jochner-Oette

**Affiliations:** 1 Physical Geography / Landscape Ecology and Sustainable Ecosystem Development, Catholic University of Eichstätt-Ingolstadt, Eichstätt, Germany; 2 Chair of Statistics and Data Analytics, School of Business, Economics and Information Systems, University of Passau, Passau, Germany

**Keywords:** Air pollution, *Betula pendula*, *Plantago lanceolata*, *Dactylis glomerata*, Urbanization gradient, Land use regression models

## Abstract

Pollen production is one plant characteristic that is considered to be altered by changes in environmental conditions. In this study, we investigated pollen production of the three anemophilous species *Betula pendula*, *Plantago lanceolata*, and *Dactylis glomerata* along an urbanization gradient in Ingolstadt, Germany. We compared pollen production with the potential influencing factors urbanization, air temperature, and the air pollutants nitrogen dioxide (NO_2_) and ozone (O_3_). While we measured air temperature in the field, we computed concentration levels of NO_2_ and O_3_ from a land use regression model. The results showed that average pollen production (in million pollen grains) was 1.2 ± 1.0 per catkin of *Betula pendula*, 5.0 ± 2.4 per inflorescence of *Plantago lanceolata*, and 0.7 ± 0.5 per spikelet of *Dactylis glomerata.* Pollen production was higher in rural compared to urban locations on average for *B. pendula* (+ 73%) and *P. lanceolata* (+ 31%), while the opposite was the case for *D. glomerata* (− 14%). We found that there was substantial heterogeneity across the three species with respect to the association of pollen production and environmental influences. Pollen production decreased for all species with increasing temperature and urbanization, while for increasing pollutant concentrations, decreases were observed for *B. pendula*, *P. lanceolata*, and increases for *D. glomerata*. Additionally, pollen production was found to be highly variable across species and within species—even at small spatial distances. Experiments should be conducted to further explore plant responses to altering environmental conditions.

## Introduction

Along urbanization gradients, high variability of different environmental factors can be observed at short distances. The variability arises from varying degrees of anthropogenic influence, such as building density and traffic volume, which are related to the Urban Heat Island effect and emission of air pollutants (McDonnell and Pickett 1990; McDonnell and Hahs 2008). As such, those gradients are also suitable for estimating the effects of increasing temperatures and pollutant concentrations using space-for-time substitution (Pickett 1989; Ziska et al. 2003). Increases in temperature have been observed in the context of climate change, e.g., the global surface temperature increased by 1.1°C (2011–2020 compared to 1850–1900) (IPCC 2022). Anthropogenic activity and related changes in the surface of the earth contribute to changes in climatic conditions and are key drivers of air pollutant concentrations (Fritsch and Behm 2021a; Kovács and Haidu 2022). Therefore, urban areas can be regarded as “harbingers” of climate change (Ziska et al. 2003) and urban–rural gradients are useful for studying ecological changes.

Pollen production is among the plant characteristics that are expected to be influenced by climate change (Damialis et al. 2019; Beggs 2021). This pollen metric is the amount of pollen produced per reproductive unit, e.g., anther, flower, or inflorescence of the plant (Galán et al. 2017). It is one of the factors influencing the amount of pollen in the air, besides the abundance of plants, prevailing weather conditions, and atmospheric transport (Agashe and Caulton 2009; Skjøth et al. 2013). In general, studies on aerobiology are highly relevant, as aeroallergens cause allergic respiratory reactions, which are increasing globally (Bergmann et al. 2016; Beggs 2021).

Pollen production has been studied for numerous species, such as the tree species *Betula* (Jato et al. 2007; Piotrowska 2008; Jochner et al. 2011; Katz et al. 2020; Kolek 2021; Ranpal et al. 2022), *Quercus* (Tormo Molina et al. 1996; Gómez-Casero et al. 2004; Charalampopoulos et al. 2013; Kim et al. 2018; Fernández-González et al. 2020; Katz et al. 2020), *Alnus* (Moe 1998), *Fraxinus* (Tormo Molina et al. 1996; Castiñeiras et al. 2019), *Acer* (Tormo Molina et al. 1996; Katz et al. 2020), *Corylus* (Damialis et al. 2011), *Cupressus* (Hidalgo et al. 1999; Damialis et al. 2011), *Olea* (Tormo Molina et al. 1996; Damialis et al. 2011), *Platanus* (Damialis et al. 2011; Katz et al. 2020), *Juniperus* (Pers-Kamczyc et al. 2020), *Pinus* (Sharma and Khanduri 2002; Charalampopoulos et al. 2013), and *Cedrus* (Khanduri and Sharma 2002) or for grass species (Subba Reddi and Reddi 1986; Prieto-Baena et al. 2003; Piotrowska 2008; Aboulaich et al. 2009; Tormo-Molina et al. 2015; Jung et al. 2018; Romero-Morte et al. 2018; Ali et al. 2022; Severova et al. 2022) and herbaceous plants such as *Artemisia* (Piotrowska 2008; Bogawski et al. 2016), *Rumex* (Piotrowska 2008), *Plantago* (Hyde and Williams 1946; Sharma et al. 1999; Piotrowska 2008; González-Parrado et al. 2015), *Parietaria* (Fotiou et al. 2011) and *Ambrosia* (Ziska and Caulfield 2000; Wayne et al. 2002; Rogers et al. 2006).

Pollen production can be influenced by environmental factors (Ziska and Caulfield 2000; Rogers et al. 2006; Damialis et al. 2011). While Barnes (2018) reported an increase in pollen production under warmer conditions, another study found the opposite (Jochner et al. 2013). Furthermore, increased carbon dioxide (CO_2_) concentrations have been reported to lead to an increase in pollen production (Ziska and Caulfield 2000; Jablonski et al. 2002; Wayne et al. 2002; Ziska et al. 2003; Rogers et al. 2006; Kim et al. 2018). For air pollutants, nitrogen dioxide (NO_2_) might reduce pollen production (Jochner et al. 2013), but also the opposite has been documented (Zhao et al. 2017). In addition, a study demonstrated that O_3_ affects plant reproduction (Darbah et al. 2008; Albertine et al. 2014). Other factors responsible for variations in pollen production include site characteristics such as stand density and exposure (Faegri and Iversen 1989), genetics (Ranpal et al. 2022), and masting including variations in resource allocation (Kelly 1994; Crone and Rapp 2014). These different findings on the effects of environmental factors on pollen production indicate that the effects may be species-specific.

In this study, we assessed the pollen production of 24 birch trees (*Betula pendula* ROTH), 82 individuals of ribwort plantain (*Plantago lanceolata* L.), and 54 individuals of orchard grass (*Dactylis glomerata* L.) along an urbanization gradient in Ingolstadt, Germany. We analyzed the relationship between pollen production and the potential influencing factors urbanization, air temperature, NO_2_, and O_3_, separately for the three species. 

## Materials and methods

### Study area

**Fig. 1:**
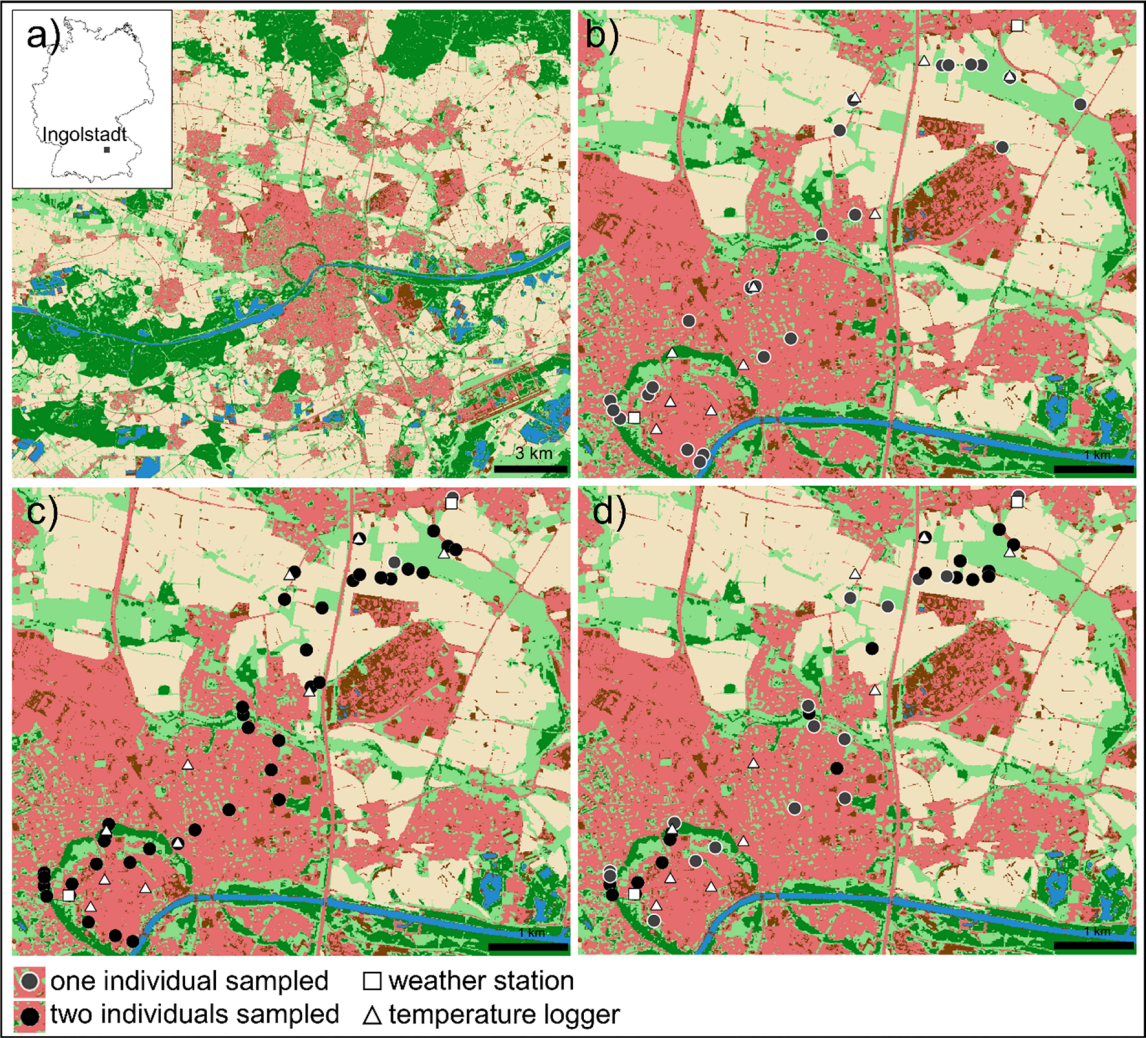
**a**) Location of the study area (black rectangle) in Germany (GeoBasis-DE/BKG 2018) and its surrounding land use **b**) sampling locations of *B. pendula*, **c**) *P. lanceolata* and **d**) *D. glomerata*. Land use: dark green—forest; light green—low vegetation; blue—water, red—built-up, dark brown—bare soil; light brown—, agriculture (mundialis 2020)

The study area is the city Ingolstadt (48.7665° N, 11.4258° E, 374 m a.s.l.) and surrounding areas, located in southern Germany (Fig. 1a), which covers an area of approx. 13,335 ha and has roughly 140,000 inhabitants (Bayerisches Landesamt für Statistik 2020). The average annual temperature is 8.9 °C, and the average annual precipitation is 712 mm (1981–2010, DWD station “Ingolstadt Flugplatz (Airport)”). The surroundings of Ingolstadt are characterized by industrial and agricultural areas, forests in the north, and riparian forests along the Danube River.

Sampling locations of the selected species *B. pendula*, *P. lanceolata*, and *D. glomerata*. were distributed along an urbanization gradient with a length of approx. 7.3 km (Fig. 1b, c, d). We focussed on these species due to their common occurrence in the study area and due to their allergological relevance (D'Amato et al. 2007; Forkel et al. 2020). The locations were characterized by different proportions of land use in their immediate surroundings.

### Fieldwork and laboratory analyses to assess pollen production

We selected 24 mature trees of *B. pendula* in the study area. For each tree, we collected a maximum of four male inflorescences (catkins) from different positions on the crown at approx. 2 m a.g.l. Catkins were harvested in February 2020, which ensured that anthesis had not started and no pollen had been released yet, following the recommendation of Damialis et al. (2011). We performed sampling and laboratory analyses according to Faegri and Iversen (1989), Damialis et al. (2011), and Ranpal et al. (2022): We measured the length and diameter of each catkin and noted the number of flowers. Catkins were put in a 10% KOH (potassium hydroxide) solution to break up plant tissue and facilitate the release of pollen grains. We used a glass rod to further crush the plant material. The solution was boiled for 10 min, and 70% Glycerol with staining Safranin was added to a volume of 20 ml to prevent pollen clumping. While stirring the solution, we used a micropipettor (Rotalibo, Carl Roth GmbH, Karlsruhe, Germany), took two samples of 10 µl each, transferred them to microscope slides, and covered them with coverslips.

For *P. lanceolata*, we sampled 82 individuals by collecting one inflorescence of each specimen in June 2020. The flower buds were fully developed but had not opened yet. We measured diameter and length of the inflorescence and determined the number of flowers. The following laboratory analyses were based on Cruden (1977) and modified to enhance the workflow. Two closed flowers of each inflorescence were detached and each of them was placed in a reaction vial, to which 10% KOH solution was added. In the next step, we used a glass rod to break up the material and added one drop of safranin and 60% glycerol to a volume of 1 ml. The tube was shaken for 10 s using a test tube shaker (Rotalibo Mini Vortex, Carl Roth GmbH, Karlsruhe, Germany) in order to mix the solution. Subsequently, we used a micropipettor as described above.

We selected 54 individuals of *D. glomerata* and collected their inflorescences with fully developed closed flowers in June 2020 (similar to *P. lanceolata*). We determined the number of flowers of each spikelet and per cm. The extraction of pollen grains and the preparation of microscope slides were performed according to the procedure used for *P. lanceolata* inflorescences.

All microscope slides were stored horizontally and counted at × 100 magnification using a light microscope (Axio Lab.A1; Zeiss, Wetzlar, Germany).

### Estimation of pollen production

The count of each microscope slide was then used to determine pollen production at different levels, depending on the species (Table 1).

**Table 1:** Overview of the different analysis levels of pollen production

Abbreviation	Description	Species
*P*_*ca*_	Pollen production per catkin	*B. pendula*
*P*_*fl*_	Pollen production per flower	*B. pendula, P. lanceolata, D. glomerata*
*P*_*infl*_	Pollen production per inflorescence	*P. lanceolata*
*P*_*sp*_	Pollen production per spikelet	*D. glomerata*
*P*_*cm*_	Pollen production per cm	*D. glomerata*

*B. pendula* pollen production was estimated per catkin *P*_*ca*_ (Damialis et al. 2011). *P*_*ca*_ was calculated by multiplying the counted pollen grains with the volume of the whole suspension and dividing it by the volume of the sample. The pollen production per flower *P*_*fl*_ was calculated by dividing *P*_*ca*_ by the number of flowers of the catkin.

*P. lanceolata* pollen production per flower *P*_*fl*_ was estimated similarly by extrapolating from the number of pollen grains contained in the analyzed volume to the whole solution. Pollen production per inflorescence *P*_*infl*_ was calculated by multiplying *P*_*fl*_ with the number of flowers.

Pollen production of *D. glomerata P*_*fl*_ was estimated in the same way as *P*_*fl*_ of *P. lanceolata*. Multiplying the number of flowers per spikelet with *P*_*fl*_ results in *P*_*sp*_*. P*_*cm*_ was calculated by multiplying *P*_*fl*_ with the number of flowers per cm.

### Temperature data

In 2019, we established a network of ten temperature loggers (Hobo Pro v2 U23-001, ONSET, Bourne, MA, USA) and two weather stations (Davis Vantage Pro2, Davis Instruments Corporation, Hayward, CA, USA) across the study area (Fig. 1). The locations were selected due to their accessibility, spatial setting in the study area and vicinity to birch trees. Temperature loggers recorded air temperature every 10 min, weather stations recorded hourly air temperature (mean, min, max). We focused on the period, which is likely to influence inflorescence and pollen formation and growth, calculated mean, and minimum and maximum temperatures. For *B. pendula*, we additionally considered the accumulated daily mean temperature (accTmean). The periods considered were June 01 to August 30, 2019, for *B. pendula* (Dahl and Strandhede 1996), and the time from the start of the vegetation period, April 02, 2020, until June 30, 2020 for *P. lanceolata* and *D. glomerata.* The start of the vegetation period was indicated when the daily mean temperature was above 5 °C for seven consecutive days (Estrella and Menzel 2006). For this regard, we assessed the temperature data of the weather station in the city center of Ingolstadt, which resulted in a start on April 02, 2020.

### Land use regression model

We used the land use regression (LUR) models of Fritsch and Behm (2021a) to obtain estimated mean annual pollutant concentrations of NO_2_ and O_3_ (µg/m^3^) for the locations where the plant material was sampled. The models are based on additive regression smoothers of spatial and structural explanatory variables and reflect the intra-urban variability (Jerrett et al. 2005) of background concentrations for the year 2019 (*B. pendula*) and 2020 (*P. lanceolata*, *D. glomerata*) at the different locations. The data used to fit the models were compiled following the description in Fritsch and Behm (2021b). We employed the most recent versions of the datasets containing the pollutant concentrations measured at the sites of the German air quality monitoring network in 2019 and 2020 (EEA 2022), land use based on the CORINE land cover classes (EEA 2020), topography (BKG 2021a), German administrative regions and population density (BKG 2021b), and the road traffic network (EuroGeographics 2022). Germany was separated into 1 × 1 km grid cells and one LUR model was estimated based on monitoring sites reflecting background concentrations for NO_2_ and O_3_. Overall, the models for NO_2_ exhibited similar properties to the ones reported in Fritsch and Behm (2021a) and highlighted agglomeration and infrastructure effects—though concentrations were generally lower. In total, air pollutant concentrations were higher for NO_2_ in more densely populated areas, while the opposite was the case for O_3_. Each model was used to predict pollutant concentration levels for the exact geographical locations at which pollen production was estimated.

### Urbanization

We employed land use data including the classes forest, low vegetation, water, built-up, bare soil, and agriculture, which are based on automatically processed Sentinel-2 satellite data and available at a resolution of 10 m (mundialis 2020). The urban index was calculated according to Jochner et al. (2012) for all sampling locations, which is defined as the share of builtup area within a 2 km radius surrounding the location. Sampling locations with an urban index of [0, 0.5] were defined as rural, and those with [0.5, 1] as urban locations.

### Statistical analyses

We analyzed the relationships between pollen production and temperature, urban index, and air pollutants by calculating Spearman’s correlation coefficient (*r*_*s*_) and investigated if there are differences in pollen production at urban and rural locations for the three plant species via a Wilcoxon rank sum test. We tested the null hypothesis that the correlation (difference) is zero for *r*_*s*_ (Wilcoxon rank sum test) and considered the correlations (differences) to be statistically significant when *p* ≤ 0.05. We additionally investigated component plus residual plots for the different environmental influences urban index, temperature (Tmean, Tmin, Tmax, accTmean), NO_2_, and O_3_ on pollen production. The plots consider the effect of one explanatory variable *X*_*j*_ on pollen production *P* at once, while controlling for all other explanatory variables ***X***_*(j)*_ based on a linear regression. Each plot shows ***X***_*j*_ (abscissa) and the partial residuals (ordinate). The latter is based on a linear regression of *P* on all environmental influences ***X*** and result from subtracting the partial effect of all environmental influences but the considered one ***X***_*(j)*_***β***_*(j)*_ from *P.* The implied partial effect is illustrated by a regression line and a univariate spline function.

We used R (4.2.2) with the packages ggplot2 (Wickham 2016), dplyr (Wickham et al. 2022), mgcv (Wood 2017), terra (Hijmans et al. 2022), sf (Pebesma et al. 2022a), stars (Pebesma et al. 2022b), npreg (Helwig 2022) and car (Fox and Weisberg 2019) for statistical analyses and visualization. For spatial analyses, we used ESRI ArcMap 10.6 and for visualization QGIS 3.14.

## Results

### Pollen production of B. pendula, P. lanceolata and D. glomerata

*P*_*ca*_ of the studied *B. pendula* trees averaged at 1.2 ± 1.0 million pollen grains and varied between 83,000 and 3.7 million pollen grains. The number of flowers per catkin averaged was at 134 ± 20, the mean length of catkins was 33.2 ± 6.4 mm, and the mean diameter was 3.7 ± 0.3 mm. Mean *P*_*infl*_ of *P. lanceolata* was 5.0 ± 2.4 million pollen grains with a range of 1.5 to 12.1 million pollen grains. We counted 73 ± 29 flowers per inflorescence on average; the mean length of the inflorescence was 13.8 ± 3.6 mm and the diameter was 6.2 ± 0.9 mm. For *D. glomerata*, the average *P*_*sp*_ was 0.7 ± 0.5 million pollen grains and ranged from 79,450 to 2.4 million pollen grains. The mean number of flowers per spikelet was 16 ± 5 (Table 2).

**Table 2:** Descriptive statistics on pollen production at different analysis levels and summary statistics on investigated plant species *B. pendula*, *P. lanceolata*, and *D. glomerata*

	Min	Median	Mean	Max	SD
*Betula pendula* (*N*_*B*_ = 24)					
Flowers per catkin	106	132	134	178	20
*P*_*fl*_	703,4	6,560	9,212	32,349	8,110
*P*_*ca*_	83,000	812,000	1,221,104	3,707,000	1,020,539
Length of catkin (mm)	22.5	32.6	33.2	49	6.4
Diameter of catkin (mm)	3.2	3.7	3.7	4.2	0.3
*Plantago lanceolata* (*N*_*P*_ = 82)					
Flowers per inflorescence	28	67	73	175	29
*P*_*fl*_	40,950	65,000	68,716	129,950	17,755
*P*_*infl*_	1,515,750	4,587,188	5,039,550	12,085,350	2,421,113
Length of inflorescence (mm)	6.6	13.2	13.8	27	3.6
Diameter of inflorescence (mm)	3.1	6.3	6.2	9.5	0.9
*Dactylis glomerata* (*N*_*D*_ = 54)					
Flowers per spikelet	7	16	16	32	5
Flowers per cm	3	12	13	24	5
*P*_*fl*_	11,350	32,606	40,407	121,150	24,339
*P*_*sp*_	79,450	487,088	660,272	2,423,000	503,273
*P*_*cm*_	52,200	397,575	538,640	2,059,550	423,721

### Pollen production and urbanization

Using the urban index to distinguish between urban and rural locations resulted in 10 rural and 14 urban locations for *B. pendula*, 46 rural and 36 urban locations for *P. lanceolata*, and 26 rural and 28 urban locations for *D. glomerata*. Considering all locations, the urban index varied between 0.30 and 0.72 (mean = 0.48).

Overall, daily mean temperature at urban locations was higher compared to rural locations, with statistically significant differences (*p* < 0.001). In 2019, during the considered period from June to August, the mean temperature at the rural location was 13.7 °C, at urban locations it was 2.1 °C higher with a mean of 15.9 °C. In the considered period in 2020 (April 02 to June 30), the temperature at rural locations averaged 13.7 °C, and at urban locations, it was 1.2 °C higher with 14.9 °C.

We found different pollutant concentrations between the years and locations. While NO_2_ concentrations ranged from 13.5 to 21.4 µg/m^3^ at the plant locations in 2019 with higher concentrations at urban (mean = 20.8 µg/m^3^) compared to rural locations (mean = 17.4 µg/m^3^). NO_2_ concentrations in 2020 were about one-third lower on average and ranged from 10.1 to 15.1 µg/m^3^ with higher concentrations in urban (mean = 13.5 µg/m^3^) compared to rural locations (mean = 12.2 µg/m^3^). In both years, the differences between the locations were statistically significant (*p* < 0.001). This also applies to O_3_ concentrations, for which urban locations averaged at 45.9 µg/m^3^ in 2019 and 44.4 µg/m^3^ in 2020, while rural locations averaged at 47.7 µg/m^3^ in 2019 and 45.0 µg/m^3^ in 2020. The maps derived from the LUR model are included in the Appendix.

**Fig. 2:**
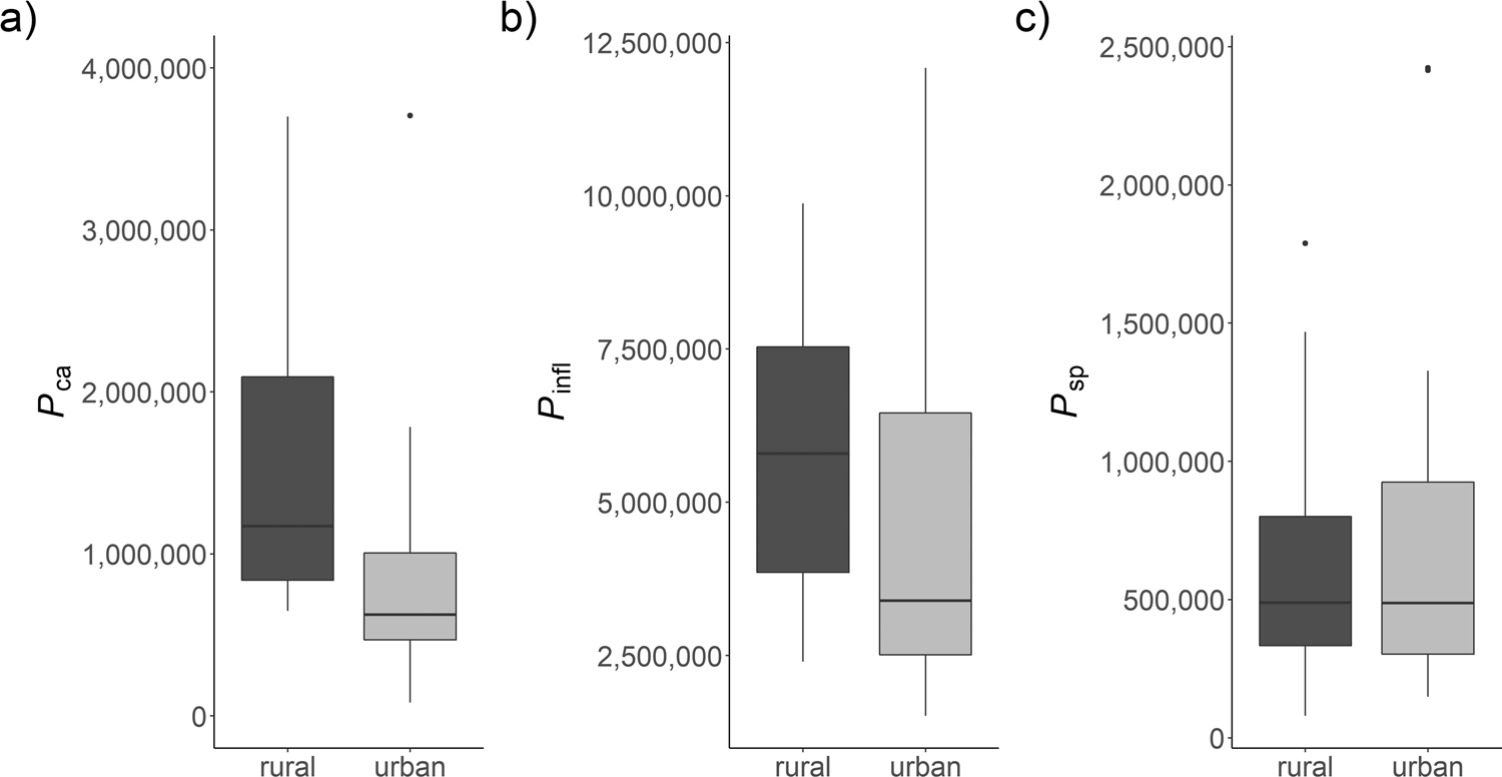
Boxplots of pollen production at rural (dark grey) and urban (grey) locations. Pollen production **a**) of *B. pendula* (*P*_*ca*_), **b**) of *P. lanceolata* (*P*_*infl*_), and **c**) of *D. glomerata* (*P*_*sp*_). Median indicated by horizontal line, interquartile range (IQR) by boxes, range of values within 1.5 times IQR by vertical lines; dots represent observations exceeding 1.5 times IQR

As illustrated in Fig. 2 and Table 3, pollen production varied between urban and rural locations for *B. pendula*: mean *P*_*ca*_ was 0.9 ± 0.9 (urban) vs. 1.6 ± 1.1 (rural) million pollen grains. This difference was statistically significant (*p* = 0.016). The mean number of flowers per catkin was 134 ± 24 (urban) vs. 135 ± 14 (rural) (no statistically significant difference, *p* = 0.618).

**Table 3:** Descriptive statistics of pollen production at urban and rural locations. *B. pendula*: Pollen grains per catkin *P*_*ca*_; *P. lanceolata*: Pollen grains per inflorescence *P*_*infl*_; *D. glomerata*: pollen grains per spikelet *P*_*sp*_

	Min	Median	Mean	Max	SD
*Betula pendula P*_*ca*_					
Urban	83,000	624,250	936,679	3,707,000	909,646
Rural	649,000	1,171,167	1,619,300	3,699,000	1,079,064
*Plantago lanceolata P*_*infl*_					
Urban	1,515,750	3,393,712	4,432,529	12,085,350	2,437,554
Rural	2,398,950	5,796,000	5,815,188	9,875,625	2,196,241
*Dactylis glomerata P*_*sp*_					
Urban	148,225	487,088	707,434	2,423,000	590,016
Rural	79,450	489,050	609,482	1,788,800	394,738

For *P. lanceolata*, mean *P*_*infl*_ was lower at urban locations (4.4 ± 2.4 million pollen grains) compared to rural locations (5.8 ± 2.2 million pollen grains). The mean number of flowers per inflorescence was higher at rural locations with 85 ± 31 flowers compared to 64 ± 24 flowers at urban locations. Both differences were statistically significant (*p* ≤ 0.003).

For *D. glomerata*, mean *P*_*sp*_ were quite similar with an average of 0.7 ± 0.6 at urban and 0.6 ± 0.4 million pollen grains at rural locations. The mean number of flowers per spikelet was 17 ± 5 flowers at urban and 15 ± 6 flowers at rural sites (*p* = 0.033).

### Connection between pollen production and environmental influences

Table 4 shows the Spearman rank correlation coefficient *r*_*s*_ of pollen production of the three analyzed species with urban index, air temperature variables, and air pollutants. While we observed negative correlations of pollen production with the urban index and all temperature variables, the strength of correlations varied across the three species. For *B. pendula*, the correlations of Tmean, Tmin and accTmean were significantly different from zero. For *P. lanceolata*, this was the case for all considered correlations (for *P*_*infl*_) and Tmean (for *P*_*fl*_), while none of the correlations were statistically significant for *D. glomerata*. For the air pollutants, correlations were negative for NO_2_ for all species besides *P*_*fl*_ for *P. lanceolata* and *P*_*sp*_ for *D. glomerata*. For O_3_, negative correlations were obtained for *P. lanceolata* and positive ones for B*. pendula* and *D. glomerata*. None of the correlations was different from zero at the considered significance level.

**Table 4:** Spearman correlation (*r*_*s*_) and *p*-values (*p*) for the Wilcoxon rank sum test that the correlation was zero between pollen production at different levels of the analyzed species and urban index, air temperature (Tmean, Tmin, Tmax, accTmean) and the air pollutants NO_2_ and O_3_. Bold letters indicate statistically significant correlations (*p* ≤ 0.05)

Urban index			Air temperature				Air pollutants	
			Tmean	Tmin	Tmax	accTmean	NO_2_	O_3_
*Betula pendula*								
*P*_*ca*_	*r*_*s*_	− 0.255	** − 0.554**	** − 0.586**	− 0.397	** − 0.549**	− 0.250	0.060
	*p*	0.229	0.005	0.003	0.055	0.005	0.239	0.780
*P*_*fl*_	*r*_*s*_	− 0.221	** − 0.533**	** − 0.569**	− 0.389	** − 0.537**	− 0.222	0.037
	*p*	0.298	0.007	0.004	0.060	0.007	0.296	0.866
*Plantago lanceolata*								
*P*_*infl*_	*r*_*s*_	** − 0.232**	** − 0.428**	** − 0.367**	** − 0.271**	**-**	− 0.134	− 0.169
	*p*	0.036	0.000	0.001	0.014	-	0.231	0.128
*P*_*fl*_	*r*_*s*_	− 0.055	** − 0.230**	− 0.186	− 0.178	-	0.004	− 0.111
	*p*	0.621	0.038	0.094	0.109	-	0.973	0.321
*Dactylis glomerata*								
*P*_*sp*_	*r*_*s*_	− 0.110	− 0.136	− 0.155	− 0.160	-	0.014	0.077
	*p*	0.430	0.325	0.262	0.249	-	0.918	0.579
*P*_*fl*_	*r*_*s*_	− 0.256	− 0.205	− 0.213	− 0.087	-	− 0.142	0.119
	*p*	0.062	0.137	0.123	0.534	-	0.305	0.391
*P*_*cm*_	*r*_*s*_	− 0.178	− 0.157	− 0.176	− 0.124	-	− 0.029	0.003
	*p*	0.197	0.258	0.202	0.370	-	0.833	0.981

**Fig. 3:**
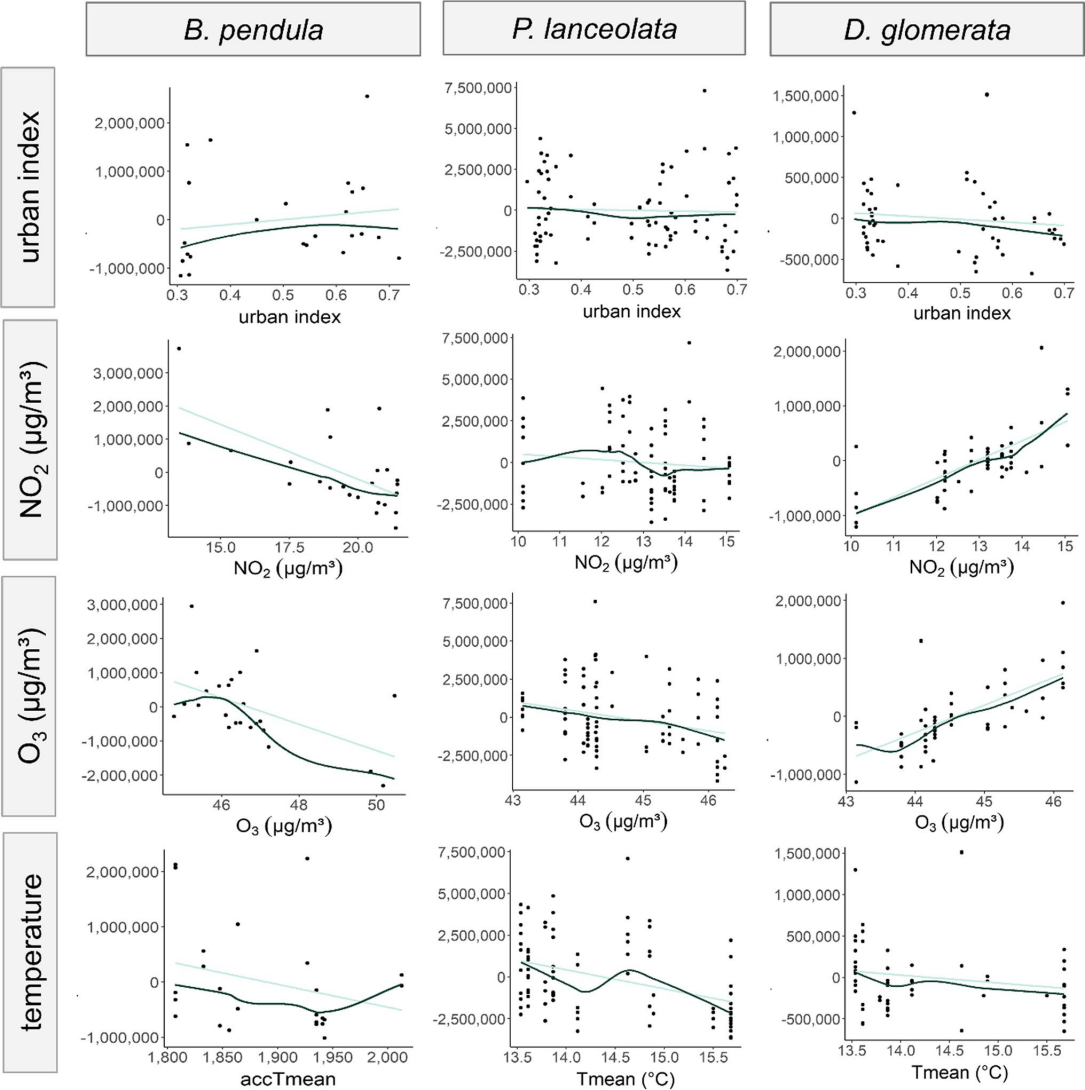
Component plus residual plots for species *B. pendula* (*P*_*ca*_), *P. lanceolata* (*P*_*infl*_) and *D. glomerata* (*P*_*sp*_). Lines of plot illustrate the effects of urban index, NO_2_, and O_3_ concentration levels (µg/m^3^) and temperature (abscissa) on pollen production when controlling for all other environmental influences via a linear regression (ordinate). Regression line is indicated by a light line, spline function by a dark line

As correlation coefficients are only suitable to assess bivariate relationships between variables, we also used the component plus residual plots displayed in Fig. 3 to investigate the connection between pollen production *P* and the *j*-th considered environmental influence, while controlling for all other influences via a linear regression. In the first line of the plot, for example, the effect of the urban index on *P* was investigated for each species, while temperature (accTmean for *B. pendula*, Tmean for *P. lanceolata*, and *D. glomerata*), NO_2_, and O_3_ are used as controls. Overall, the figure mostly confirms the findings reported in Table 4 and indicates that increasing temperature has a negative effect on pollen production. The degree of urbanization has a positive effect on pollen production of *B. pendula* and negative effects on *P. lanceolata* and *D. glomerata*. For the air pollutants, negative effects are indicated for *B. pendula* and *P. lanceolata* and positive ones for *D. glomerata*. When considering the other available temperature variables for the three plant species (Tmin, Tmax), the results remain qualitatively identical. The plots also indicate that a linear regression line approximates the relationship between pollen production and environmental influences reasonably well, as the spline function and the regression line largely coincide.

## Discussion

### Pollen production of *B. pendula*, *P. lanceolata*, and *D. glomerata*

This study contributes to the knowledge of pollen production of the allergenic species *B. pendula*, *P. lanceolata*, and *D. glomerata* and its connection with environmental influences. The chosen study area reflected urban–rural differences, as indicated by the differences between the sites regarding temperature and air pollutant concentrations. Urban–rural differences in temperature varied between 1.2 and 2.1°C. NO_2_ concentrations differed between the years, illustrating the effect of COVID-19-related lockdowns on air pollution, which was documented for Germany (Balamurugan et al. 2021; Cao et al. 2022).

We investigated the pollen production of 24 *B. pendula* trees. Our results for *P*_*ca*_ averaged at 1.2 ± 1.0 million pollen grains (range from 83,000 to 3.7 million pollen grains). Other recent studies examining *P*_*ca*_ of *B. pendula* reported 1.7 ± 1.3 million pollen grains (Ranpal et al. 2022), 0.6 ± 0.6 million pollen grains (Kolek 2021), 10 million pollen grains (Piotrowska 2008), or between 4.8 and 8.2 million pollen grains (Jato et al. 2007). For other *Betula* species such as *B. papyrifera*, 24.3 million pollen grains per catkin were estimated (Katz et al. 2020).

Pollen production of *P. lanceolata* averaged at 5.0 ± 2.4 million pollen grains with a range of 1.5 to 12.1 million pollen grains per inflorescence in this study (sample size *N*_*P*_ = 82). González-Parrado et al. (2015) estimated a mean of 5.3 million pollen grains, with a minimum of 1.8 and a maximum of 9 million pollen grains. Piotrowska (2008) estimated *P*_*infl*_ of *P. major* in a similar range with 6.3 million pollen grains.

We documented a mean of 40,407 ± 24,339 pollen grains per flower of *D. glomerata* (sample size *N*_*D*_ = 54). Tormo-Molina et al. (2015) reported an average of 5,431 pollen grains per flower, ranging between 2033 and 9600, in Badajoz, Spain. The mentioned results vary greatly, possibly caused by the great difference in geographic location.

Several factors must be considered when comparing estimates of pollen production, one being the used sampling and laboratory methods. The pollen produced by an inflorescence or flower can be determined by drying and weighing the flowers or inflorescence (Jochner et al. 2011; Beck et al. 2013; Jung et al. 2018) or the extraction and determination of the pollen amount using light microscopy (Cruden 1977; Damialis et al. 2011). The latter method is often applied in research (e.g., Damialis et al. 2011; Ranpal et al. 2022, 2023) and was also used in this study. However, when extrapolating from one analyzed level to others, e.g., from a flower to the whole tree, small errors can alter the values to a great extent. In addition, the application of the method is easier for some species than for others. For example, single flowers in the correct phenological stage could easily be extracted from the inflorescences of *P. lanceolata*. In the case of *D. glomerata*, it turned out to be a bit more demanding due to the structure of the plant, spikelets, and flowers. In addition, the estimation of pollen production is labor-intensive, which underlines the need for an automated procedure in pollen detection and counting, which has been the focus of recent studies (e.g., Ali et al. 2022).

### Pollen production, urbanity, and environmental influences

We found a higher pollen production at rural locations for the two studied species, *B. pendula* (*P*_*ca*_) and *P. lanceolata* (*P*_*infl*_) compared to urban locations. In addition, we found a higher number of flowers per inflorescence of *P. lanceolata* and of flowers per spikelet of *D. glomerata*. All three described differences were statistically significant. There are only few studies that have analyzed differences in pollen production in regards to urbanization, while there are a few more that examined relationships between pollen production and environmental factors. Jochner et al. (2011) assessed the pollen production of 26 *B. pendula* trees in urban and rural areas of Munich, Germany, in 2009. The authors detected higher pollen values in the rural area at the start of flowering (assessed using the weighing method). In Augsburg, Germany, pollen production of *B. pendula* was investigated by Kolek (2021) who reported higher pollen production with increasing urbanization. Ziska et al. (2003) assessed pollen production of common ragweed along an urbanization gradient in Maryland, USA. They found that ragweed produced more pollen in urban than in rural locations, which is different from our findings on the three studied species.

We analyzed the connection between environmental influences and pollen production by considering bivariate Spearman rank correlations and component plus residual plots. Our results indicate a negative effect of temperature on pollen production for *B. pendula* und *P. lanceolata* and that the functional relationship can be approximated by linear regression. This indicates a decrease in pollen production with increasing temperatures (when leaving all other observable and unobservable influences constant). The relationship between the air pollutants NO_2_ and O_3_ and pollen production was found to be negative for *B. pendula* and *P. lanceolata*, while the opposite was the case for *D. glomerata*. This result implies that these air pollutants affect pollen production – even when accounting for the other considered environmental influences. In general, our results indicate that the effect of environmental influences on pollen production is species-specific. Jochner et al. (2013) reported negative correlations of birch pollen production with temperature, atmospheric NO_2_ as well as foliar concentrations of the nutrients potassium and iron, but with temperature identified as the most important influencing factor. Furthermore, Kolek (2021) found no correlation between *P*_*ca*_ and cumulated minimum temperature of the summer months (June to August) of the previous year. In addition, there were no significant correlations between *P*_*ca*_ and O_3_ or NO_2_. In our study, correlations between *P*_*ca*_, *P*_*fl*_, and the cumulative temperature were, however, significant and negative. Darbah et al. (2008) studied the effect of elevated O_3_ on *Betula papyfera* and reported higher production of catkins along with a decrease in catkin length, diameter, and mass. Studying *Ambrosia artemisiifolia,* Zhao et al. (2017) found increased pollen and decreased seed production under elevated NO_2_ concentrations.

We applied a land use regression (LUR) model to generate data on the air pollutants NO_2_ and O_3_ at a fine spatial scale. Our model took air quality data from the monitoring stations in Germany, land cover, topography, population density, and road traffic into account. However, like all modeling approaches, LUR models have limitations (Hoek et al. 2008), i.e., the influence of individual pollutants is not considered separately and modeling accuracy strongly depends on the accuracy of the input variables. Despite these limitations, LUR models are a cost-effective tool to obtain data on air quality, when the equipment of a large study area with air monitoring devices is too resourceful and when background concentrations of air pollutants are of interest. However, we encourage incorporating site-specific pollution data, as well as meteorological data, i.e., air temperature, and precipitation, in further studies. These data cannot only be used for examining links to pollen production, but also for the comparison of data derived from LUR models.

By studying pollen production along an urbanization gradient, we made use of the space-for-time approach. However, investigations along with other gradients should be considered for future studies. One approach was recommended by Tito et al. (2020), who suggested using altitudinal gradients as “natural laboratories” and transplanting and translocating species from different locations on the gradient to others. Such experiments could also consider all plant characteristics and the plant’s physiological performance, i.e., visual parameters of the plants such as the amount of flowers or foliage, but also other characteristics such as the allergenicity of pollen.

Besides temperature and air pollution, other site conditions might modify plant growth, which was also suggested by González-Parrado et al. (2015). These site conditions include the proximity to agricultural land to which fertilizers are presumably applied. In addition, better soil quality with higher soil moisture would be expected in more rural settings. When considering the immediate surroundings of our study sites, in some cases *P. lanceolata*, the species with higher pollen production at rural sites, was growing in areas close to agricultural land. In addition, some sites in urban areas did not seem favorable for plant growth due to the vicinity to roads and possible exposure to waste and high nitrogen input (Allen et al. 2020). In contrast, we did not observe higher pollen production of *D. glomerata* in rural areas. This might indicate that this grass species is not benefitting as much from the mentioned site conditions as *P. lanceolata*. Furthermore, a study examining *Juniperis communis* pollen reported that nutrient availability had an impact on the development of pollen grains (Pers-Kamczyc et al. 2020). Their results on pollen production and pollen quality suggest that plants growing in nutrient-rich settings produce a higher amount of pollen to compensate for the lower quality of pollen grains.

Our results revealed differences in the number of flowers between urban and rural locations for *P. lanceolata* and *D. glomerata*. Similar results were found in the case of *Brassica rapa* (Rivkin et al. 2020), as there were significantly fewer flowers on plants in urban sites. Rivkin et al. (2020) suggested that reason for this might be the exposure to exhaust fumes emitted by cars that might lead to foliar damage, affecting photosynthetic capacity and growth rates.

While our findings give new insights into the possible effects of changing temperatures and air pollution on pollen production, shortcomings have to be accounted for, such as the pollen production data that were based on one year. Conducting this investigation over more years would lead to more robust results, and in addition, influences on pollen production such as masting would be considered. Masting is the phenomenon of trees producing a high number of flowers and seeds in one year, which is then followed by a period of lower seed production (Herrera et al. 1998; Ranta et al. 2005, 2008; Crone and Rapp 2014). The duration of this period can vary, for *Betula* it has been observed to be every two (Latałowa et al. 2002) or three years (Detandt and Nolard 2000). However, there have been no studies on the masting behavior of birch trees in cities, so it is not possible to assess the extent to which masting plays a role in our study. This phenomenon could explain the differences in pollen production estimates between our study and those from the literature (e.g., Jato et al. 2007; Piotrowska 2008; Kolek 2021). Therefore, we strongly suggest the investigation of pollen production over several years in further studies.

## Conclusion

In this study, we showed variations in pollen production of three allergenic species along an urbanization gradient. Pollen production for two species was overall higher in rural compared to urban locations and we found negative relationships with temperature for *B. pendula* and *P. lanceolata*, and positive relationships with NO_2_ and O_3_ for *D. glomerata*. Further studies should *inter alia* focus on the physiological performance of trees growing in urban areas, which might give a hint for explaining their behavior related to pollen production. Furthermore, additional locations representing semi-urban sites would support the continuous representation along the gradient. This is essential for drawing conclusions and may allow more profound predictions related to the future effects of climate change on pollen production. In order to identify single influencing factors, a combination of experiments in a controlled environment with field research should be considered.

## Data Availability

Data sharing is not applicable, we therefore did not include a statement.

## Appendix

Maps illustrating the LUR-derived concentrations for NO_2_ and O_3_ for the years 2019 and 2020 for the study area (black rectangle represents the extent of Fig. 1a) and surrounding regions.
